# Systematic Review on Individualized Versus Standardized Parenteral Nutrition in Preterm Infants

**DOI:** 10.3390/nu15051224

**Published:** 2023-02-28

**Authors:** Walter Mihatsch, Miguel Ángel Jiménez Varas, Lucia Lorenzino Diehl, Virgilio Carnielli, Rahel Schuler, Corinna Gebauer, Miguel Sáenz de Pipaón Marcos

**Affiliations:** 1 Department of Pediatrics, Ulm University, 89075 Ulm, Germany; 2 Department of Health Management, Neu-Ulm University of Applied Sciences, 89231 Neu-Ulm, Germany; 3 Health Sciences Library, Hospital Universitario La Paz, 28046 Madrid, Spain; 4 Department of Neonatology, Department of Pediatrics, Hospital Universitario La Paz, Universidad Autonoma de Madrid, 28046 Madrid, Spain; 5 Department of Mother and Child Health, Division of Neonatology, G. Salesi Children’s Hospital, 60123 Ancona, Italy; 6 Department of Odontostomatologic and Specialized Clinical Sciences, Polytechnic University of Marche, 60020 Ancona, Italy; 7 Department of General Pediatrics and Neonatology, Justus-Liebig-University, 35392 Giessen, Germany; 8 Department of Neonatology, Leipzig University Hospital, 04103 Leipzig, Germany

**Keywords:** parenteral nutrition, preterm infant, individualized, standardized, safety, outcome, necrotizing enterocolitis, sepsis, mortality, growth, protein, nutrition

## Abstract

The need for high quality evidence is recognized for optimizing practices of parenteral nutrition (PN). The purpose of the present systematic review is to update the available evidence and investigate the effect of standardized PN (SPN) vs. individualized PN (IPN) on protein intake, immediate morbidities, growth, and long-term outcome in preterm infants. A literature search was performed on articles published in the period from 1/2015 to 11/2022 in PubMed and Cochrane database for trials on parenteral nutrition in preterm infants. Three new studies were identified. All new identified trials were nonrandomized observational trials using historical controls. SPN may increase weight and occipital frontal circumference gain and lower the value of maximum weight loss. More recent trials suggest that SPN may easily increase early protein intake. SPN may reduce the sepsis incidence, but overall, no significant effect was found. There was no significant effect of standardization of PN on mortality or stage ≥2 necrotizing enterocolite (NEC) incidence. In conclusion SPN may improve growth through higher nutrient (especially protein) intake and has no effect on sepsis, NEC, mortality, or days of PN.

## 1. Introduction

Parenteral Nutrition (PN) is a lifesaving therapy for preterm infants. PN is indicated when oral or enteral nutrition is not possible, insufficient, or contraindicated in order to avoid undernutrition and related adverse consequences. The nutrient stores of very low birth weight (VLBW, birth weight <1500 g) and extremely low birth weight (ELBW, birth weight <1000 g) preterm infants are low. Bridging PN ensures adequate fluid intake and nutrient supply for weight gain and possibly neurodevelopmental long-term outcome. VLBW infants are vulnerable to postnatal growth failure because the gut is immature, and provision of nutrients is challenging. The evidence showing the beneficial effects of enhanced PN to VLBW infants is accumulating. Providing amino acids and energy immediately after birth with PN is a standard practice to promote positive nitrogen retention [1]. PN can be provided as a standard pre-specified formulation or individually prescribed solutions.

Individual prescriptions for PN are ordered and prepared every 24–48 h. The main advantage of individually prescribed PN is that it is tailored to suit a specific patient, thereby assuring the best possible nutrition and biochemical control [2]. However, several limitations such as errors, stability issues and risk of infections have been reported [2]. Batch-produced standardized PN bags can be readily available as ward stocks in neonatal intensive care units, enabling initiation of early PN immediately after delivery of a premature infant. Moreover, standard PN solutions incorporate expert nutritional knowledge and support [2].

Both techniques have been employed. Based on a low level of evidence, limited randomized controlled trials (RCTs) and some retrospective observational studies, in 2018 the combined working group on pediatric parenteral nutrition of ESPGHAN, ESPEN, ESPR, and CSPEN conditionally recommended that standard PN solutions (SPN) should generally be used over individualized PN solutions (IPN) in the majority of pediatric and newborn patients, including VLBW premature infants [3]. This recommendation has recently been endorsed by the UK National Guideline Alliance [4]. In addition, the combined working group recommended that individually tailored PN solution should generally be used when the nutritional requirements cannot be met by the available range of standard PN formulations (i.e., in very sick and metabolically unstable patients such as those with abnormal fluid and electrolyte losses; and in infants and children requiring PN for prolonged periods such as those with short bowel syndrome [3]. This recommendation will always be valid.

The optimal PN management, SPN or IPN, is still controversial. The provision of PN is highly complex, requiring high-quality pharmacy aseptic manufacturing services. The proposed practical benefits of SPN were as follows:

- Improved patient safety (minimization of procedural incidents),
- Provision of higher early intakes of amino acids and glucose, and better calcium phosphate ratio during the first week of life [5,6],
- Prevention of ordering and compounding errors—due to the complexity of the supply chain much of the variations in actual nutrient intake are unintended [7],
- Improved pharmaceutical control of the physicochemical stability and aseptic manufacturing [8] by large scale industrial production and
- Reduction in costs [9].

Variation in PN macronutrient intake (glucose, protein or lipid intake) also results from differences in nutritional policy [10] and use of central, in contrast to peripheral, venous catheters which enable the use of more concentrated PN solutions. PN is recognized as a high risk and complex treatment. There is a need to compare outcomes including adverse events (sepsis, due to a less complex aseptic preparation, and mortality), growth (including weight gain) and protein intake, particularly influential on growth, as a surrogate measure of all other PN components, where the evidence base is still incomplete and questioned. One would expect to achieve better nutritional goals with IPN, tailored to individual needs. The ready-to-use triple-chamber SPN solutions with the option to add additional nutrients such as vitamins, trace elements, or amino acids are possibly easier to use by less experienced doctors. The aim of the present systematic review was to update the available evidence and investigate the effect of SPN vs. IPN on protein intake, immediate morbidities, growth and long-term outcome in preterm infants.

## 2. Materials and Methods

### 2.1. Design

This review was designed to update the 2018 conditional recommendation of the combined working group on pediatric parenteral nutrition of ESPGHAN, ESPEN, ESPR, and CSPEN that standard PN solutions (SPN) should generally be used over individualized PN solutions (IPN) in the majority of preterm infants [3] using standard methods [11] and the Preferred Reporting Items for Systematic Reviews and Meta-Analyses (PRISMA) criteria [12,13]. The level of evidence of eligible studies and the degree of recommendation were assessed following the recent guideline approach [1,3]. The research questions were defined following the PICO framework [14] (Table 1). Primary outcomes were defined as protein intake, immediate in-hospital complications such as mortality, sepsis incidence, necrotizing enterocolitis (NEC) incidence, duration of PN (days), growth, and neurodevelopmental long-term outcome. The NEC data were defined and extracted as stage ≥2 NEC [15]. Growth was assessed as weight gain in g/kg/d during the study period, weight standard deviation score (SDS) at discharge, head circumference (HC) SDS at discharge, or weight and occipitofrontal circumference (OFC) SDS change from birth to 36 weeks postmenstrual age.

### 2.2. Search Strategies

A literature search on articles published from 1/2015 to 11/2022 was performed in PubMed and Cochrane databases for clinical trials on parenteral nutrition in preterm infants. The literature search started in 2015 because the 2018 guideline was based on a systematic literature search up to 2015 and some trials published in 2016. The search strategy for each electronic database is given in Table S1.

### 2.3. Selection Criteria

Inclusion criteria included all clinical trials published in any language, but providing an English language abstract. All retrieved records were imported into EndNote X9, and duplicates were removed automatically and by manual checking. The titles and abstracts of the outputs were screened independently by two reviewers (MS and WM) to select the potential trials. Then, the full text of each potential trial was further assessed for eligibility. The reviewers also screened the reference lists of eligible trials to identify further relevant eligible trials. All eligible trials were finally included after discussions between the reviewers. Two reviewers (MS and WM) extracted the following information: Author, year of publication or update, country or region, population, intervention, comparison, and outcomes. The GRADE approach was used to assess the quality of evidence and to interpret findings. Authors evaluated the level of evidence (LoE), the grade of recommendation (GOR), and the form of recommendation as described previously [11]. The SIGN classification was used to assign both the evidence level and the recommendation grade. The scales used to evaluate LoE, GOR, and form of recommendation are summarized in Table 2, Table 3 and Table 4 [11,16].

### 2.4. Statistics

Outcomes for categorical data are presented as odds ratios with respective 95% confidence intervals. For continuous data, the weighted mean difference with 95% confidence interval was used. The treatment effects of individual trials and heterogeneity between trial results were examined by inspecting the forest plots. The impact of heterogeneity in any meta-analysis was assessed using a measure of the degree of inconsistency in the studies’ results (I-squared statistic). A random effects model for metanalyses was used. Review Manager (RevMan) Version 5.4 software, Copenhagen: The Nordic Cochrane Centre, The Cochrane Collaboration, 2014 was used for data analysis.

## 3. Results

**Figure 1:**
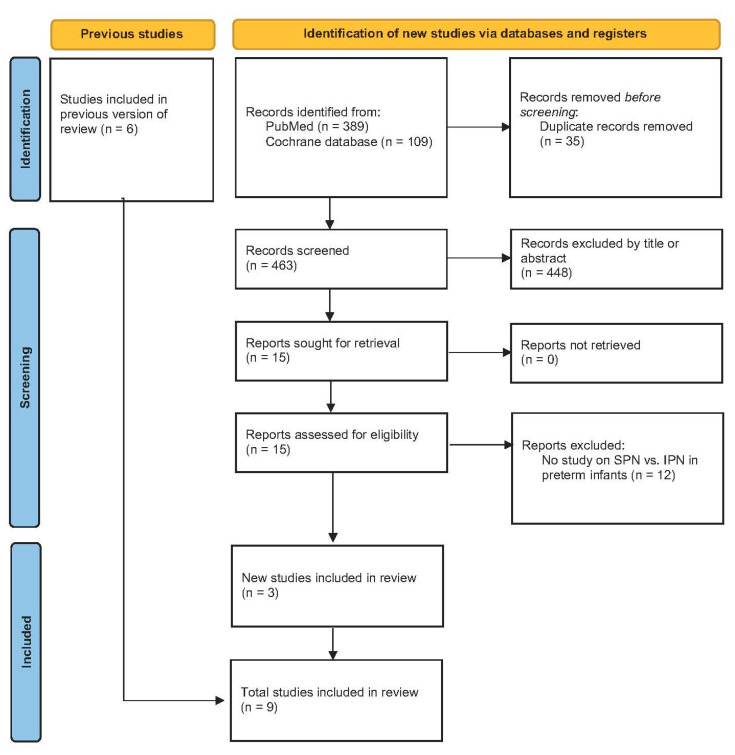
PRISMA flow diagram. (Standardized parenteral nutrition (SPN), individualized parenteral nutrition (IPN)).

PRISMA flow diagram is given in Figure 1. Altogether 498 potential hits were found in PubMed and Cochrane database. After removal of duplicates 463 potential papers were assessed by title and/or abstract. Fifteen potential papers were assessed for eligibility and 12 were excluded. Three new studies [17,18,19] (Table 5) were identified. Together with the six studies already included in the previous ESPGHAN review [20,21,22,23,24,25] (see Table S2) now nine clinical trials were included in the present review. The innovation of two of the previous studies was individualization of PN by pharmacists (IPN with pharmaceutical individualization) in contrast to standardized PN approaches monitored and organized by neonatologists [20,24]. More recent observational trials studied individualization by neonatologists (IPN) in contrast to standard PN bags (SPN).

All new identified trials were nonrandomized observational trials using historical controls and therefore provide a low level of evidence (LoE 2-) only.

- The study by Evering et al. was designed as a retrospective cohort study comparing IPN (2011) to partially SPN (2012) and completely SPN (2014) consequently [17]. The partial SPN group was not included in the present review.
- The study by Immeli et al. was designed as a retrospective cohort study comparing IPN (2005–2007) to two-in-one SPN (2008–2009), a second two-in-one SPN (2010–2011), and finally a triple-chamber-SPN (2012–2013) consequently [19]. For analysis of sepsis and NEC incidence the SPN groups were merged. For analysis of first week protein intake the triple-chamber SPN group only was used.
- The retrospective observational trial by Morgan et al. compares the data of two RCTs performed in the same department from 2004–2006 and 2009–2012 [18]. The first study was a RCT of normal vs. high nutrient IPN, the second a RCT of SPN vs. high nutrient SPN. The forest plots provide two comparisons based on this data. Standard SPN vs. standard IPN (Morgan 2019 part A) and high nutrients SPN vs. high nutrients IPN (Morgan 2019 part B).

**Figure 2:**
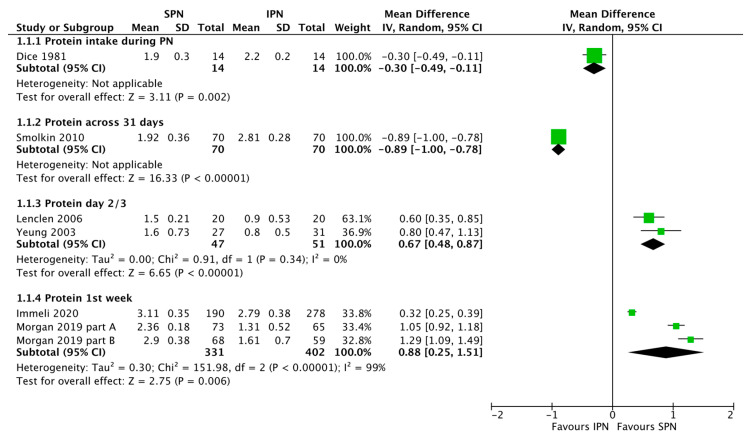
Forest plot on the effect of standardized parenteral nutrition (SPN) vs. individualized parenteral nutrition (IPN) on protein intake. Green squares represent individual studies, diamonds represent subgroup analyses.

**Figure 3:**
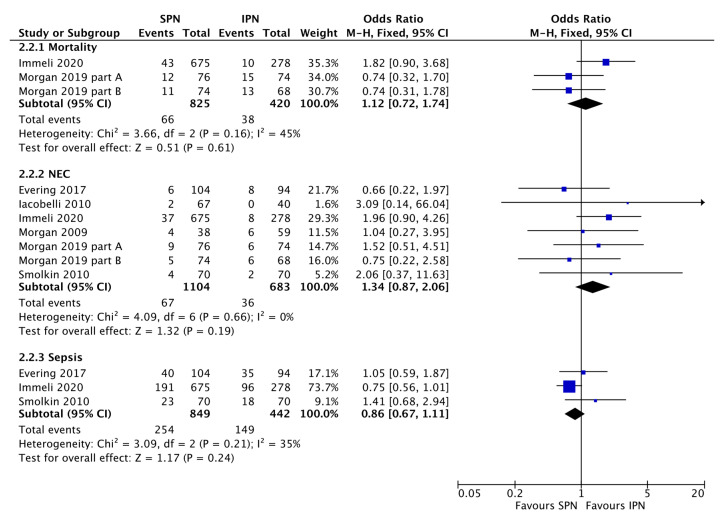
Forest plot on the effect of standardized parenteral nutrition (SPN) vs. individualized parenteral nutrition (IPN) on mortality, necrotizing enterocolitis (NEC) incidence, and sepsis incidence. Blue squares represent individual studies, diamonds represent subgroup analyses.

**Figure 4:**
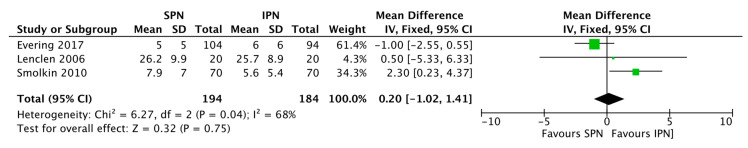
Forest plot on the effect of standardized parenteral nutrition (SPN) vs. individualized parenteral nutrition (IPN) on duration of PN (days). Green squares represent individual studies, the diamond represents the subgroup analysis.

**Figure 5:**
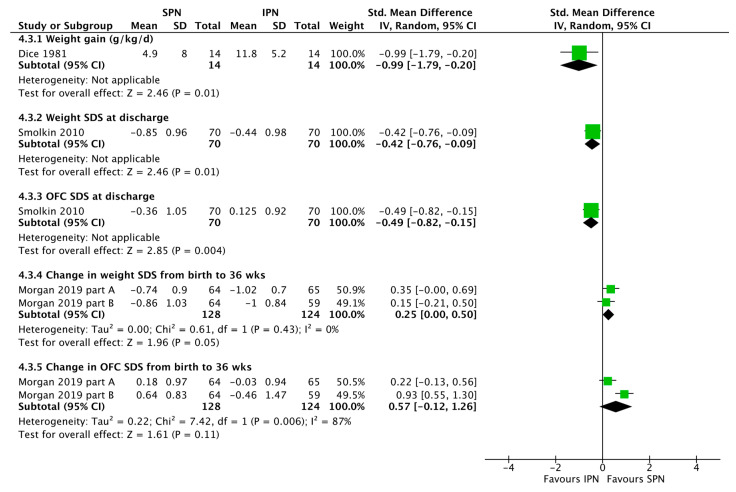
Forest plot on the effect of standardized parenteral nutrition (SPN) vs. individualized parenteral nutrition (IPN) on growth. Green squares represent individual studies, diamonds represent subgroup analyses.

Forrest plots of meta-analyzed outcomes are given in Figure 2, Figure 3, Figure 4 and Figure 5. More recent trials suggest that SPN may easily increase early protein intake (Figure 2). It is important to appreciate the heterogeneity of the reported protein intake data. The length of the reported protein intake periods varied. Therefore, Figure 2 presents protein intake data for various time periods as reported in different studies. Within these time periods SPN infants received SPN.

The large observational study by Immeli et al. [19], especially suggests that SPN may reduce the sepsis incidence but overall no significant effect was found (Figure 3). There was also no significant effect of standardization of PN on mortality or stage ≥2 NEC incidence (Figure 3). SPN does not necessarily reduce the duration of PN (Figure 4). SPN may increase in-hospital weight and OFC gain, lower the value of maximum weight loss, and increase mean cumulative weight gain during the first three weeks (Figure 5 and Evering et al. [17]).

There was no data regarding the long-term outcome of preterm infants meeting the inclusion criteria for this review.

## 4. Discussion

PN plays an important role in the nutritional support of preterm infants. However, PN practice is usually described as often with insufficient intakes, poor growth and adverse events. Both SPN and IPN are currently used in preterm clinical practice. A SPN is a pre-defined formulation made to a set composition that does not vary. Their efficient and safe use in pediatric patients—as in adults in whom they have been used for more than 10 years—is based on the tenet that many patients with a similar condition have comparable nutritional needs allowing their management with one or several balanced SPN formulations. Prepared in advance by a hospital pharmacy or an external compounding center, SPN bags are rapidly available when indicated.

### 4.1. Conclusion 1: The LoE of the Available Studies Comparing SPN vs. IPN Is Very Low (LoE1- and LoE 2-)

Overall, the evidence provided by the studies available in the present systematic review is very low (one study LoE 1-, eight studies LoE 2-). All new identified trials and all but one of the trials of the 2018 review were nonrandomized observational trials using historical controls. There is only one underpowered randomized controlled trial, conducted more than 40 years ago (1981) when the “standard of care” was very different from current clinical practice and standard bags were not yet commercially available. Based on poor allocation concealment (alternate assignment to treatments) in the previous guideline the trial was downgraded to LoE 2+. A total of 28 preterm infants were assigned by alternation to IPN or SPN. IPN including pharmaceutical individualization increased amino acid (AA) intake (2.2 ± 0.2 vs. 1.9 ± 0.3 g/kg/d, *p* < 0.01), non protein energy intake (63.0 ± 7.0 vs. 53.0 ± 6.0 kcal/kg/d, *p*< 0.001) and finally weight gain (11.8 ± 5.2 vs. 4.9 ± 8.0 g/kg/day, *p* < 0.02) [20]. Of note, glucose manipulations in the SPN group required diluting the standardized TPN formulation with a glucose solution and consequently reducing the intake of other nutrients including amino acids. Of note, the study by Morgan et al., 2019 is not an RCT comparing SPN vs. IPN. It compared two recent previous RCTs (one IPN and one SPN) each compared standard (control) and high (intervention) parenteral protein and energy dosage regimens [23].

### 4.2. Conclusion 2: IPN with Pharmaceutical Individualization, May Increase Protein Intake, and the Use of Ready to Use SPN Bags May Facilitate Early Achievement of Protein Needs

#### 4.2.1. IPN with Pharmaceutical Individualization

The concept that IPN with pharmaceutical individualization is superior to SPN organized by neonatologists is supported by the above-mentioned randomized trial [20] and by one of the observational trials [24]. In a cohort of 140 VLBW infants born between 2000 and 2007, significantly higher daily glucose, AA, lipid intakes and achievement of complete enteral intake in a shorter time were found with IPN together with pharmaceutical individualization [24].

#### 4.2.2. Ready to Use SPN Bags

Several more recent observational trials suggest that ready to use SPN solutions may facilitate early (first week) achievement of protein needs (Figure 2). Similar results regarding higher amino acid intake with SPN vs. IPN were reported by Lenclen 2006 and Yeung 2003. In a prospective cohort study, Yeung studied 58 neonates, with a gestational age <33 weeks, and found that neonates with SPN received more AA each day and more Ca and P on the third day of life [25]. Lenclen et al. studied 40 neonates with a gestational age <32 weeks and found that SPN was superior in terms of early glucose provision and AA on day 3 of life (less variation in PN protocol and earlier onset compared with personalized PN) and a better Ca/P ratio in the first week of life [22]. In 2010, Iacobelli et al. studied 107 newborns with a gestational age < 33 weeks and found that those receiving standard PN had significantly higher glucose, AA, lipids, sodium, and magnesium [21].

The study by Immeli et al. was designed as a retrospective cohort study comparing IPN (2005–2007) to two-in-one SPN (2008–2009), a second two-in-one SPN (2010–2011), and finally a triple-chamber-SPN (2012–2013) consequently [19]. With regard to the present review, for analysis of first week protein intake, Group 1 (IPN) vs. Group 4 (SPN), the licensed triple-chamber manufactured by the industry, were used. SPN manufactured by the industry under regulated quality standards, was associated with improved protein intake, and the protein target was more likely to be achieved [19].

Finally, Morgan 2019 compares the data of two RCTs performed in the same department 2004–2006 and 2009–2012 [18]. The first study was a RCT of normal vs. high nutrient IPN, the second a RCT of SPN vs. high nutrient SPN. The forest plots provide two comparisons based on this data. Standard SPN vs. standard IPN (Morgan 2019 part A) and high nutrients SPN vs. high nutrients IPN (Morgan 2019 part B). SPN was introduced within 6 h of birth with an amino acid starting dose of 1.8 g/kg/day, while IPN was introduced within 24 h (where possible) with a starting dose of 1 g/kg/day. SPN can improve PN efficiency and compliance with guidelines. The earlier and faster introduction of SPN compared with IPN resulted in much higher target protein intakes for the SPN groups in the first 5 days of life. The first factor that may improve the protein intake is the lower level of deviation from the protocol. Mean gestational ages were lower in the IPN groups.

Altogether, expert individualization by a pharmacy may improve PN nutrient intake in preterm infants [20,24]. However, all the observed effects on nutrient intake of SPN vs. historical IPN controls entirely depend on historical changes in SPN and IPN composition and nutritional targets. Of note, the SPN arm of the Immeli et al., 2020 study (Figure 2) was the only subgroup which, on average, approached current preterm infants target protein needs.

### 4.3. Conclusion 3: Reflecting the Above-Mentioned Data on Protein Intake, IPN with Pharmaceutical Individualization Was Associated with Better in-Hospital Growth [20,24] Whereas Most Recent Observational Studies Using Ready to Use SPN Solutions Observed Better Growth Than Historic Controls without Pharmaceutical IPN Individualization (Figure 5)

#### 4.3.1. Pharmaceutical IPN Individualization

Consistently, the data reconfirmed the positive association between protein intake and growth [28]. Higher protein intake was associated with improved weight gain. Therefore, pharmaceutical IPN individualization improved weight gain in the randomized trial by Dice et al. (11.8 ± 5.2 vs. 4.9 ± 8.0 g/kg/day, *p* < 0.02) [20] and in the observational trial by Smolkin et al. (Figure 5). The latter study in a cohort of 140 low birth weight infants born between 2000 and 2007, showed significantly greater weight gain SDS during the 1st week (*p* =0.036) and the 1st month of life (*p* = 0.0004), and higher discharge weight SDS (*p* = 0.012) and OFC SDS (*p* = 0.006) in IPN.

#### 4.3.2. Ready to Use SPN

The available data is very limited. SPN vs. historic control IPN without pharmaceutical individualization was associated with improved weight gain and head growth until 36 weeks in the observational study by Morgan et al. (Figure 5) [18]. SPN reduced the value of maximum weight loss [17,21] and increased mean cumulative weight gain during the first three weeks [17] in two further studies.

However, the currently available commercial SPN solutions may not be the optimum approach. Commercial SPN may need an additional amino acid supply to achieve recommended target intakes and consequently adequate growth defined as intrauterine growth velocity [5].

### 4.4. Conclusion 4: SPN vs. IPN Did Not Reduce Sepsis or NEC Incidence, Mortality, or PN Duration

Beyond improved nutrient intake, the umbrella aim in introduction of SPN is to improve patient safety (minimization of hospital associated incidents) possibly at the cost of increased PN solution wastage [29]. Due to the complexity of the supply chain much of the variations in actual nutrient intake are unintended and SPN may contribute to prevent ordering and compounding errors [7]. Several in-hospital SPN production quality and stability control studies have been published more recently [30,31,32,33]. In a longitudinal quality improvement study in pediatric patients and infants > 1500 g standardizing TPN and transitioning to electronic ordering was associated with reduced ordering errors, reduced processing time and most importantly substantially reduced number of blood draws [34]. In junior residents, introduction of SPN reduced medication errors and improved time management [35]. Commercial SPN bags in addition, guarantee sterility.

However, SPN did not significantly prevent hospital associated complications (Figure 3). Especially supported by one very large observation trial [19] it is tempting to interpret the data that there may be a trend that SPN prevents nosocomial sepsis, but there was also a trend in the opposite direction with regard to NEC and no effect on mortality or duration of PN (Figure 3). SPN still requires carful nutritional individualization and increasing the number of local SPN solutions did not reduce the number of fluid imbalances, electrolyte derangements and glucose derangements [36].

### 4.5. Recommendations

#### 4.5.1. Recommendation 1

Standard PN Solutions (SPN) Should Generally Be Used over Individualized PN Solutions (IPN) in the Majority of Preterm Infants, Including VLBW Premature Infants (LoE 2-, GPP, Conditional Recommendation).

#### 4.5.2. Recommendation 2

Individually Tailored PN Solution should Generally Be Used When the Nutritional Requirements Cannot Be Met by the Available Range of Standard PN Formulations (i.e., in Very Sick and Metabolically Unstable Patients Such as Those with Abnormal Fluid and Electrolyte Losses; and in Infants and Children Requiring PN for Prolonged Periods Such as Those with Short Bowel Syndrome (LoE 2, RG B, Strong Recommendation for) [3].

It has been estimated that the majority of preterm infants receiving PN via central catheters may actually be treated by SPN, although, the optimum composition is not known [37,38]. SPN can improve PN efficiency and compliance with guidelines. No significant harmful effects have been found by the present systematic review. Therefore, the present review reconfirms the 2018 ESPGHAN, ESPEN, ESPR, and CSPEN recommendation that SPN solutions should generally be used over IPN solutions in the majority of pediatric and newborn patients, including VLBW premature infants [3].

#### 4.5.3. Recommendation 3

Adequately Powered Randomized Controlled Trials Based on Up-To-Date Parenteral Nutrition Recommendations Are Required to Evaluate the Real Clinical Benefits of SPN vs. IPN (LoE 1, GOR A, Strong Recommendation).

### 4.6. Strengths and Limitations

Strengths of the present review are its exhaustive search of the available literature, reproducibility, systematic assessment of evidence and grading of recommendations. However, there are important limitations. Neonatology is rapidly developing and changing. E.g., nutritional recommendations changed several times within the last 20 years. Using historical controls rather than contemporaneous controls carries a high risk of bias and grossly limits the level of evidence. Improvements in nutritional status may rather be a consequence of increased nutritional awareness than a consequence of new developed PN approaches. The observed effects may have been noticed by chance and adequately powered, randomized controlled trials based on up-to-date parenteral nutrition recommendations are urgently required to evaluate the real clinical benefits of SPN bags. Given current parenteral nutrition recommendations a considerable proportion of infants of the reported trials are undernourished. In addition, the composition of SPN and IPN nutritional regimens and consequently the observed nutritional status, varied across and within the different studies, limiting the validity of the results of the meta-analyses. In the clinical experience of the authors, one SPN does not fit all preterm infants. The smaller the infants are, the more often adjustments are required, commercial SPN bags are more expensive than hospital pharmacy produced SPN bags, and finally commercial SPN bags still require pharmacy-based adjustments (e.g., supplementation of vitamins and trace elements).

## 5. Conclusions

We conclude that SPN may improve growth through higher nutrient (especially protein) intake and has no effect on sepsis, NEC, mortality, or days of PN. These observations may have been noticed by chance and adequately powered randomized controlled trials are required to evaluate the real clinical benefits of SPN.

## Figures and Tables

**Table 1:** Research questions defined following the PICO framework [14].

PICO Framework	
Population	Infants born preterm, up to 28 days after their due birth date
Intervention	Any standardized approach to providing parenteral nutrition
Comparison	Any individualized parenteral nutrition solutions (bespoke prescriptions)
Outcomes	Protein intake Adverse events Sepsis NEC, stage ≥ 2 Mortality Duration of hospital stay Growth/anthropometric measures Neurodevelopmental outcomes

**Table 2:** Rating scheme for the strength of the evidence [11,16].

Level of Evidence (LoE)	Type of Evidence
1++	High quality meta-analyses, systematic reviews of randomized controlled trials (RCTs), or RCTs with a very low risk of bias
1+	Well conducted meta-analyses, systematic reviews of RCTs, or RCTs with a low risk of bias
1−	Meta-analyses, systematic reviews of RCTs, or RCTs with a high risk of bias
2++	High quality systematic reviews or case control or cohort studies. High quality case control or cohort studies with a very low risk of confounding or bias and a high probability that the relationship is causal
2+	Well conducted case control or cohort studies with a low risk of confounding or bias and a moderate probability that the relationship is causal
2−	Case control or cohort studies with a high risk of confounding or bias and a significant risk that the relationship is not causal
3	Non-analytic studies, e.g., case reports, case series
4	Expert opinion

**Table 3:** Rating scheme for the strength of the recommendation [11,16].

Grade of Recommendation (GOR)	Level of Evidence
A	At least one meta-analyses, systematic reviews or RCT rated as 1++, and directly applicable to the target population; or a body of evidence consisting principally of studies rated as 1+, directly applicable to the target population and demonstrating overall consistency of results
B	A body of evidence including studies rated as 2++, directly applicable to the target population; or a body of evidence including studies rated as 2+, directly applicable to the target population and demonstrating overall consistency of results; or extrapolated evidence from studies rated as 1++ or 1+
0	Evidence level 3 or 4; or extrapolated evidence from studies rated as 2++, 2+, or 2−
GPP	Good practice points: Recommended best practice based on the clinical experience of the guideline development group

**Table 4:** Forms of recommendation [11,16].

Judgement	Recommendation
Undesirable consequences clearly outweigh desirable consequences	Strong recommendation against
Undesirable consequences probably outweigh desirable consequences	Conditional recommendation against
Balance between desirable and undesirable consequences is closely balanced or uncertain	Recommendation for research and possibly conditional recommendation for use restricted to trials
Desirable consequences probably outweigh undesirable consequences	Conditional recommendation for
Desirable consequences clearly outweigh undesirable consequences	Strong recommendation for

**Table 5:** New identified studies. All new studies are nonrandomized trials using historical controls (LoE 2-). (Standardized parenteral nutrition (SPN), individualized parenteral nutrition (IPN)).

Study	Population	Intervention	Comparison	Outcomes	Comments
Morgan [18] 2019 UK	292 VLBW infants Mean BW 905 g Mean GA 26 wks	SPN Standard SPN of RCT 2 High target SPN of RCT 2	IPN Standard IPN of RCT 1 High target IPN of RCT 1	Protein intake Gain in weight Gain in OFHC No of infants with supplementary K or P infusion	SPN infants from a RCT in SPN [26] vs. historic control infants from a RCT in IPN [27]
Evering [17] 2017 The Netherlands	N = 198 Mean GA: 205 days (SD 26.5)	SPN (*n* = 104) NEOmix—contained per 100 mL: 66 kcal with, 2.6 g protein, 2.0 g triglycerides, 8.9 g gluc, and fixed other nutrients	IPN (*n* = 94) Variable amounts of energy, protein, triglycerides, glucose, and other nutrients	Weight gain/loss TPN duration Days in NICU Mortality Sepsis	A group of 101 infants receiving partially standardized bags was excluded
Immeli [19] 2020 Finland	N = 953 VLBW infants 28,4 wks 1060 g	SPN: Numeta G13E	IPN: days 1–2 in-hospital SPN afterwards IPN	Energy intake Protein intake Length of stay mortality	Retrospective cohort study 2005–2013 IPN vs. 2 in 1 vs. 3 chamber bags

## Data Availability

Data are available on request.

## Supplementary Materials

The following supporting information can be downloaded at: https://www.mdpi.com/article/10.3390/nu15051224/s1, Table S1: Search strategy; Table S2: Table of clinical trials already included in the 2018 review.
