# Supplementary material for: Systematic Review on Individualized Versus Standardized Parenteral Nutrition in Preterm Infants
Source: Nutrients. 2023 Feb 28;15(5):1224. doi: 10.3390/nu15051224 (PMC10005430; doi:10.3390/nu15051224)
Supplement: Supplementary file 1 [file nutrients-15-01224-s001.zip › Suppl 1.pdf]

Supplemental material 1

Table S1: SEARCH STRATEGY:

Database	Search Strategy	Studies (N)
**PUBMED**	(("infant"[MeSH Terms] OR "infant*"[Title/Abstract] OR "infancy"[Title/Abstract] OR "newborn*"[Title/Abstract] OR "new born*"[Title/Abstract] OR "newly born"[Title/Abstract] OR "baby*"[Title/Abstract] OR "babies"[Title/Abstract] OR "neonat*"[Title/Abstract] OR "low birth weight"[Title/Abstract] OR "low birthweight"[Title/Abstract] OR "ELBW"[Title/Abstract] OR "Preterm"[Title/Abstract] OR "pre term"[Title/Abstract] OR "prematur*"[Title/Abstract] OR "LBW"[Title/Abstract] OR "VLBW"[Title/Abstract]) AND ("Parenteral Nutrition"[MeSH Terms] OR "parenteral nutrition*"[Title/Abstract] OR "parenteral feeding*"[Title/Abstract] OR "intravenous feeding*"[Title/Abstract]) AND ("standar*"[Title/Abstract] OR "individual*"[Title/Abstract] OR "custom*"[Title/Abstract] OR "personal"[Title/Abstract])) AND (("2015/01/01"[Date - Publication] : "2022/11/30"[Date - Publication]))	389
**CENTRAL** (Cochrane Library)	#1 MeSH descriptor: [Infant] explode all trees<br>#2 (infant* OR infancy OR newborn* OR 'new born*' OR 'newly born' OR baby* OR babies OR neonat* OR 'low birth weight' OR 'low birthweigh' OR ELBW OR Preterm OR 'pre term' OR prematur* OR LBW OR VLBW):ti,ab<br>#3 #1 OR #2<br>#4 MeSH descriptor: [Parenteral Nutrition] explode all trees<br>#5 ('Parenteral Nutrition' OR 'Parenteral Feeding*' OR 'Intraveous feeding*'):ti,ab<br>#6 (standar* OR individual* OR custom* OR personal):ti,ab<br>#7 #4 OR #5<br>#8 #3 AND #7<br>#9 #8 AND #6 with Publication Year from 2015 to 2022, in Trials	109
