# Supplementary material for: Systematic Review on Individualized Versus Standardized Parenteral Nutrition in Preterm Infants
Source: Nutrients. 2023 Feb 28;15(5):1224. doi: 10.3390/nu15051224 (PMC10005430; doi:10.3390/nu15051224)
Supplement: Supplementary file 1 [file nutrients-15-01224-s001.zip › Suppl 2.pdf]

Table S2: Table of clinical trials already included in the 2018 review

Study	Population	Intervention	Comparison	Outcomes	Comments
LoE 1+/++: High quality RCTs or systematic reviews of high quality RCTs					
none					
Level 1-: Low quality RCTs or systematic reviews of all RCTs					
Level 2+: Nonrandomized, contemporaneous controls					
Dice[1] 1981	N=28<br>Mean BW: 1109g (SD 211.37)<br>Mean GA: 31 weeks (SD 1.52)	SPN (n=14)<br>Solutions prepared in the pharmacy, physicians were allowed to make essential glucose and electrolyte manipulations.<br>Standard solutions were available as either 10% or 13% dextrose.	IPN (n=14)<br>Individual requirements were determined by the physician and pharmacist.	Weight gain<br>Protein intake<br>Non-protein calorie intake	Babies assigned to SPN or IPN alternately
LOE 2-: Nonrandomized, historical controls					
Iacobelli. 2010[2]<br>France	N = 107<br>Mean BW: 1175g (SD 333.48)<br>Mean GA: 29 weeks (SD 1.77)	SPN (n=67)<br>Designed to provide identical initial dosage and proportional increase as indicated by the written protocol of the unit.<br>Solutions were commercially batch-produced following criteria of Fasonut Laboratories (Montpellier, France)	IPN (n= 40)<br>Prescriptions were developed using a computer system which calculated nutrient volumes according to data entered by the physician	Weight loss<br>Sepsis<br>NEC<br>Amino acid intake<br>Glucose intake<br>Lipid intake<br>Energy intake	Study conducted over 2 time periods
Smolkin[3] 2010<br>Israel	N = 140<br>Mean BW: 1285g (SD 298.58)<br>Mean GA: 29 weeks (SD 1.83)	SPN (n=70)<br>Five pre-set formulations were available with various glucose concentrations (2.5, 5, 7.5, 10 and 11%) and AA concentrations ranging from 1.5 to	IPN (n=70)<br>A standard formula was started until tailored IPN became available (glucose 7.5-11%), which could be up to 48hours.<br>IPN was adjusted daily for water, glucose, AA,	Weight gain<br>Weight at discharge<br>Head circumference<br>Duration of stay<br>Sepsis<br>Energy intake<br>Protein intake<br>Fat intake<br>Glc intake	

		2g/100ml of PN.	lipids, electrolytes, vitamins and trace elements.		
Morgan[4] 2009 UK	N = 118 Mean BW: birth weight: 930g (SD 222.85) Mean GA: 27 weeks (SD 1.51) D	SPN (n=38) The macronutrient content did not differ to the IPN. The aqueous content was concentrated and the remaining fluid provided by dextrose. The aqueous solution had a standard electrolyte content with three different options: no electrolytes, maintenance electrolytes, and additional sodium.	IPN (n= 59) The protocol aimed to start PN within 24 hours, starting at 1g/kg/day protein/lipid, increasing to 2g/kg/day for another 48 hours, with a maximum 3g/kg/day protein/lipid). Electrolyte content was individually prescribed, if deficiencies were identified the IPN was changed.	Calorie intake Protein intake	
Lenclen[5] 2006 France	N = 40 Mean GA: 28 weeks (SD 2.42) Mean BW: 886g (SD 203.60)	SPN (n=20) Prescription of PN was based on 3 solutions of predefined composition designed with reference to published evidence. Solutions were prepared in the hospital pharmacy.	IPN (n=20) Individualized following recommendations of the unit, using a standard prescribing protocol. Solutions were prepared by the nurses in the department.	Weight gain Duration of PN Non-protein energy intake AA intake Glucose intake	
Yeung[6] 2003 Australia	N = 58 Mean BW:1101g (SD 293.47) Mean GA:28 weeks (SD 1.93)	SPN (n=27) Formulations were batch produced as two solutions Solution A: Glucose 125g/L, AA 24.5g/L, Sodium 8.0mmol/L Solution B: Glucose 100g/L, AA 24.5g/L, Sodium 25mmol/L,:	IPN (n=31) Formulations were determined according to the neonatologist's discretion, based on morning serum biochemical data	Gluc intake AA intake	

References

1. Dice JE, Burckart GJ, Woo JT, Helms RA. Standardized versus pharmacist-monitored individualized parenteral nutrition in low-birth-weight infants. *Am J Hosp Pharm.* 1981;38(10):1487-1489.
2. Iacobelli S, Bonsante F, Vintejoux A, Gouyon JB. Standardized parenteral nutrition in preterm infants: early impact on fluid and electrolyte balance. *Neonatology.* 2010;98(1):84-90.
3. Smolkin T, Diab G, Shohat I, et al. Standardized versus individualized parenteral nutrition in very low birth weight infants: a comparative study. *Neonatology.* 2010;98(2):170-178.
4. Morgan C, Badhawi I, Grime C, Herwitker S. Improving early protein intake for very preterm infants using a standardised concentrated parenteral nutrition formulation. *e-SPEN.* 2009;4:e324-328.
5. Lenclen R, Crauste-Manciet S, Narcy P, et al. Assessment of implementation of a standardized parenteral formulation for early nutritional support of very preterm infants. *Eur J Pediatr.* 2006;165(8):512-518.
6. Yeung MY, Smyth JP, Maheshwari R, Shah S. Evaluation of standardized versus individualized total parenteral nutrition regime for neonates less than 33 weeks gestation. *J Paediatr Child Health.* 2003;39(8):613-617.
